# Addressing the Role of Conformational Diversity in Protein Structure Prediction

**DOI:** 10.1371/journal.pone.0154923

**Published:** 2016-05-09

**Authors:** Nicolas Palopoli, Alexander Miguel Monzon, Gustavo Parisi, Maria Silvina Fornasari

**Affiliations:** Departamento de Ciencia y Tecnología, Universidad Nacional de Quilmes, CONICET, Roque Saenz Peña 352, Bernal, (B1876BXD), Buenos Aires, Argentina; Universita' di Padova, ITALY

## Abstract

Computational modeling of tertiary structures has become of standard use to study proteins that lack experimental characterization. Unfortunately, 3D structure prediction methods and model quality assessment programs often overlook that an ensemble of conformers in equilibrium populates the native state of proteins. In this work we collected sets of publicly available protein models and the corresponding target structures experimentally solved and studied how they describe the conformational diversity of the protein. For each protein, we assessed the quality of the models against known conformers by several standard measures and identified those models ranked best. We found that model rankings are defined by both the selected target conformer and the similarity measure used. 70% of the proteins in our datasets show that different models are structurally closest to different conformers of the same protein target. We observed that model building protocols such as template-based or *ab initio* approaches describe in similar ways the conformational diversity of the protein, although for template-based methods this description may depend on the sequence similarity between target and template sequences. Taken together, our results support the idea that protein structure modeling could help to identify members of the native ensemble, highlight the importance of considering conformational diversity in protein 3D quality evaluations and endorse the study of the variability of the native structure for a meaningful biological analysis.

## Introduction

In the last years, recognition of the strong relationship between function and structure has driven a steady improvement in algorithms and methods to predict protein structure. These efforts are justified by the difficulties in experimentally determining the structure of several proteins and the enormous amount of biological information made available when protein structure is known. It is important to recognize that protein function is more related with protein dynamism than with any single structure [1,2]. Following this view, the native state of proteins is not represented by a unique structure and is better described by an ensemble of conformers in equilibrium. The need for considering different conformations in order to explain biological function could be generalized to most proteins [3]. A classic example is hemoglobin, whose function could not be fully understood without considering its different conformers, namely the tense (T) and relaxed (R) forms displaying low and high affinity for oxygen respectively [4]. The structural differences between conformers usually range from relative movements of whole domains to small-scale changes like the displacement of secondary structural elements and the rearrangement of loops, but could also be as specialized as the rotation of a single side chain [5–7]. A thorough comparison of experimentally solved conformers with identical sequences has a distribution of Root Mean Square Deviation (RMSD) values centered in 0.3Å with a large positive skew and maximums above 20Å [8]. These structural changes define conformations with varied effects on affinity and movement of ligands (substrate, product, modulators) required to sustain the biological function. For example, many ligands move from the surface of the molecule through pockets and tunnels to reach cavities containing active site residues, a process mainly regulated by a molecular gate switching between different conformations [9]. This population of conformers, mingled in a dynamic equilibrium, defines as a whole the structural basis of protein function.

The structural differences between conformers characterize the so-called conformational diversity of the protein. In spite of the time that has passed since Monod postulated the importance of the different conformations to explain protein function[10], this concept has only recently become central to explain a vast and increasing list of different biological processes. Besides the classical models explaining cooperativism and allosteric effects in proteins and enzymes through conformational diversity[4], the concept has also been employed to describe numerous processes such as enzyme catalysis [11] and promiscuity [12], protein-protein recognition [13] and signal transduction[14], mechanisms of disease-related mutations[15] and immune escape[16], the origin of neurodegenerative diseases [17], protein evolutionary rates[18], conformer-specific substitution patterns[19], the origins of new biological functions [20] and co-evolutionary measurements between residues [21]. Lately, conformational diversity has been considered in new computational tools for ligand docking and protein-protein interaction predictions [22], the development of biologically active ligands[23] and the evaluation of protein structure models[24]. Bioinformatics is a fast-paced but relatively novel discipline and despite the great progress in structural modeling and quality assessment over the last decade[25], one of the next steps needed to improve functional characterization is related with the development of methods that can explicitly predict or take into account the conformational ensemble of the native state. This is of major importance for many quality assessment protocols that heavily rely on structural comparisons (like those applied in the CASP competition [26]) and in the derivation of several model validation programs [27,28]. The results obtained by these structure-based strategies may thus be biased by the use of unique target structures selected as representatives of the whole native state. Moreover, the way of measuring protein structure similarity will influence the evaluation of decoy quality and alter their ranking, as typically used metrics like root mean square deviation (RMSD), global distance test score (GDT) and template modeling score (TM-score) [29] display different sensibilities to structural variation.

In this work we studied how a set of protein models obtained by different 3D structure prediction methods reproduce the conformational ensemble of selected proteins. For 91 proteins used as targets in different 3D prediction experiments, we collected the proposed computational models and evaluated their structural similarity against alternative PDB structures[30] of the same protein taken from CoDNaS database [31,32], which provides an empirical sample of the native conformational diversity of each protein. Our results confirm the possibilities of state-of-the-art in silico protein structure modeling methods to sample the native conformational variability of proteins and highlight the need for developing new structural quality assessment methods to address conformational diversity in order to improve our understanding of the complex relationship between protein structure and function.

## Methods

### Dataset construction

We collected protein structure models from three public datasets: the whole set of CASP experiments from CASP3 to CASP10 (http://predictioncenter.org/) and two decoy sets obtained with Rosetta@home[33] (‘All Atoms decoy set’ accessed on January 2015 at http://depts.washington.edu/bakerpg/) and QUARK[34]. Our analysis was limited to the subset of target proteins with observed conformational diversity as identified by cross-linking with CoDNaS database[31,32]. Initially, and from all available CoDNaS conformers of a protein, we only considered those structures with 100% sequence identity to the target and showing ligand changes, post-translational modifications or different oligomeric states as possible causes of the observed conformational diversity. We measured all pairwise Cα-RMSD (hereafter RMSD for simplicity) between CoDNaS conformers using MAMMOTH[35] and selected the most distant pair of structures (representing the maximum conformational diversity observed for the protein) for further considerations. In the case of NMR solved structures, all models in the PDB file were treated as separate conformers. From every decoy set we discarded those structures with more than five consecutive missing residues or less than 80% coverage to the corresponding target. This process resulted in the generation of a compiled dataset of 182 conformers from 91 target proteins (with a total of 127121 decoys) gathering 47 (19361), 23 (2760) and 21 (105000) targets from the CASP, Rosetta and QUARK experiments respectively.

### Structural comparisons

For every target, each of both conformers derived from CoDNaS were used as reference to calculate the TM-score and GDT_TS using TM-score [29] and the RMSD with MAMMOTH against all the corresponding decoys. Aiming to recognize members of the same fold, only RMSD-based comparisons with a MAMMOTH alignment score above the cutoff [-ln(*E*)>4] were considered. This cutoff is based on the statistical significance of the expected random value *E* for that superposition. All remaining decoy-conformer pairs were ranked according to the measures above and the similarity of the decoy rankings against each of the two available conformers were studied using Spearman’s rank correlation coefficient.

### Conformational diversity studies

The selected conformers for each target protein were studied using different structural parameters in an attempt to characterize the biological relevance of their conformational diversity. In particular, we estimated the rASA (relative Accessible Surface Area) using DSSP [36] which was then normalized by the ASA in tripeptides according to [37] and finally used to derive a ΔrASA between conformers. We applied the programs MOLE [38] and FPocket [39] to predict the presence of tunnels, pockets and cavities and estimate their structural changes between conformers. Visual inspection of the structures and a review of relevant bibliography allowed us to identify interesting candidates for discussion.

## Results and Discussion

### Characterization of conformational diversity in selected proteins

Our studies were based on the analysis of 91 proteins for which there is experimental evidence of conformational diversity in the native state (Table 1). Since these proteins were used in different 3D modeling experiments, a number of structural models or decoys are publicly available and can be used to assess if protein modeling would account for conformational diversity. From all possible target proteins in the datasets of origin, we only took those included in CoDNaS database. CoDNaS is a database of putative conformers: it stores redundant collections of structures of the same protein sequence determined experimentally under different biological conditions. These conditions, such as pH, presence of ligands, oligomeric state, post-translational modifications and so forth, could be associated with the changes observed among structures of the same protein, thus providing an interesting biological background to characterize their conformational diversity.

**Table 1:** Datasets used in the analysis. Shown are the number of proteins and the overall number of decoys in each dataset, the minimum and average TM-score (in TM-score units), the minimum and average GDT_TS (in GDT_TS units) for all targets in each dataset and their maximum and average Cα-RMSD (in Å) calculated likewise. Datasets and statistics are available as S1 Dataset and as a compressed file from our website at http://ufq.unq.edu.ar/sbg/files/Palopoli-Monzon_2016_SI.tar.gz.

Dataset	Proteins	Decoys	GDT_TS (min)	GDT_TS (avg)	TM-score (min)	TM-score (avg)	Cα-RMSD (max)	Cα-RMSD (avg)
**CASP 3–10**	47	19361	0.15	0.89	0.30	0.91	3.26	1.20
**QUARK**	21	2760	0.30	0.81	0.27	0.80	1.40	0.82
**Rosetta@home 2007**	23	105000	0.44	0.80	0.40	0.79	2.87	1.64

**Fig 1:**
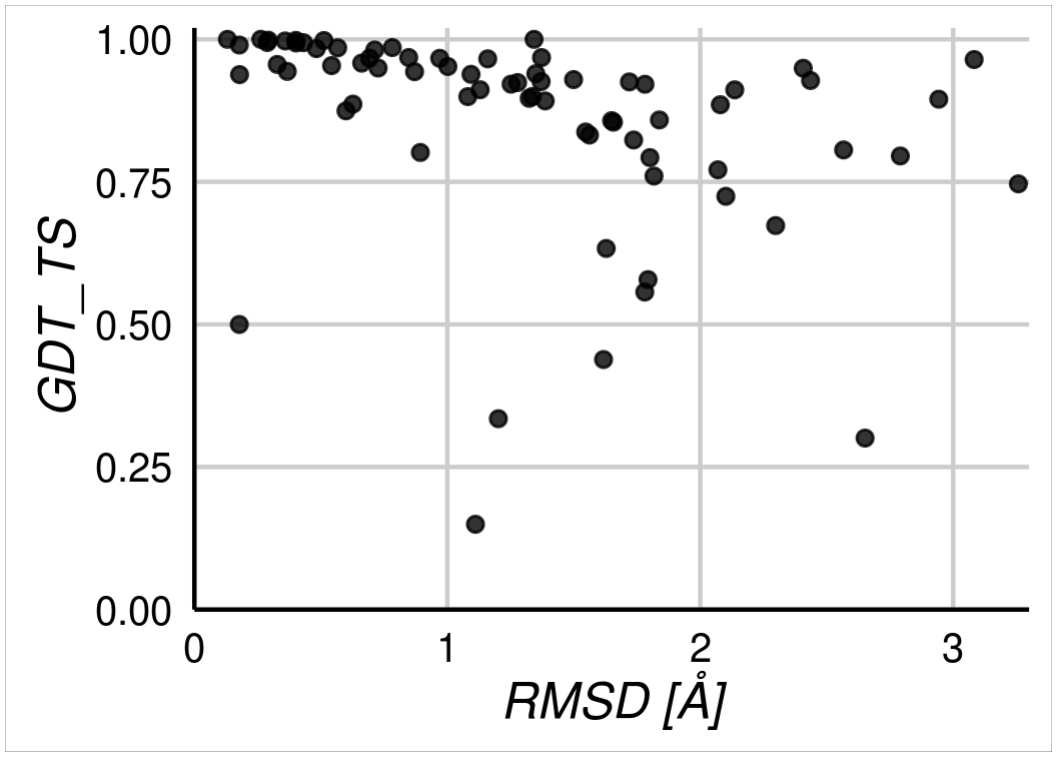
Comparison of GDT_TS against RMSD, calculated for pairs of conformers with maximum conformational diversity for each protein in our dataset. Pairs were taken from the CoDNaS database of different structures (from available PDB files) for each represented protein. RMSD scores are expressed in Å while GDT_TS values are normalized to the range [0, 1].

For each of the 91 selected proteins we performed pairwise structural comparisons of all conformers derived from CoDNaS. Unsurprisingly, even subtle structural perturbations associated with the presence of a bound ligand can have serious biological implications. S1 Appendix shows some examples of targets in our dataset evidencing different degrees of conformational diversity derived from changes in experimental conditions. Although CoDNaS database lists as many alternative structures of a protein as can be found in the PDB, many conformers bear no recognizable differences among them and thus are not of much benefit for studies on conformational diversity. In order to limit the redundancy of the data but at the same time to sample widely the extension of the native state, for each protein in our dataset we only analyzed the pair of conformers showing the higher RMSD between them. While different structural similarity measures have been developed[40] here we considered GDT_TS, TM-score and RMSD (Cα-RMSD) as indicators of conformational diversity. In Fig 1 we show the correlation of GDT_TS and RMSD values between all pairs of maximally distant conformers (TM-score displayed a close to perfect correlation with GDT_TS, as evidenced in S1 Fig, so it was left out of the discussion for simplicity). A marked structural similarity is generally reflected by both high GDT_TS and low RMSD values. While GDT_TS normally performs better at detecting if two structures have the same fold, RMSD can be more sensitive to movements in loops and tails and, as a consequence, it should be more adequate to detect local structural differences between native conformers of the same protein. Owing to this sensitivity, RMSD has been used in most of the studies of protein conformational diversity[41][42][21]. Since MAMMOTH calculates RMSD together with a statistical evaluation of its reliability[35] we decided to focus on the RMSD as the principal measure of similarity.

Table 1 indicates average and maximum RMSD and GDT_TS values observed for each protein. Most of the pairs of conformers with maximum conformational diversity show structural changes of different degrees. The subset of structure pairs that differ in the presence of ligands (42% of the total) displayed a distribution of maximum RMSD per dataset with a mean value of 1.17Å (max = 4.0Å, min = 0.02Å). This value is slightly smaller than observed for those maximum pairs differing in post-translational modifications (6.50%) or oligomeric states (26%), with mean maximum RMSD of 1.18Å (max = 3.84 Å, min = 0.14Å) and 1.39Å (max = 3.98Å, min = 0.09Å), respectively. The most diverse set of conformers (25%) corresponded to the alternative NMR structures (mean RMSD = 2.14Å). Thus, structural variability ranges from localized and small changes in loops or secondary elements to the relative displacement of entire domains. These movements are sometimes associated with a variation in the relative accessible surface area (rASA), although this metric shows no correlation with the protein-specific degree of conformational diversity as measured by the RMSD (S2 Fig). This lack of correlation could suggest, for example, that the major global rearrangements normally associated with high ΔrASA values cannot be directly linked to a broadening of the native structure landscape. Instead, local changes may play an important functional role in native structural diversity. In agreement, comparing predictions obtained by FPocket and MOLE for the pairs of conformers in our dataset, we found that 78% of the proteins change the number and volume of their cavities and 57% differ in the quantity of tunnels connecting cavities with the surface (S3 Fig). All these structural variations between conformers define, and may be responsible for, their different biological activities. Thus, none of the native conformers could be used by itself to provide a thorough explanation of the biological function of the protein, as this relies on the interplay among all conformers in the ensemble[9,14,20,22,43].

Following from above, and due to the importance of analyzing structural differences between conformers as a proxy to understand protein function, the next section is focused on the possibility of applying common 3D modeling tools to reproduce the conformational diversity of selected protein targets.

### Conformational diversity and 3D prediction

Started 20 years ago, the CASP initiative has established itself as a landmark community-wide effort for the assessment of protein structure prediction methods[44,45]. 3D prediction is blindly evaluated in CASP by having undisclosed, experimental knowledge of the tertiary structure of a target protein and performing structural comparisons between the target and the models submitted by participating groups. Best models are then automatically determined according to rankings of structural similarity and other evaluation measures[46]. A similar strategy is taken by other large-scale benchmarking experiments (e.g. CAPRI [47]).

**Fig 2:**
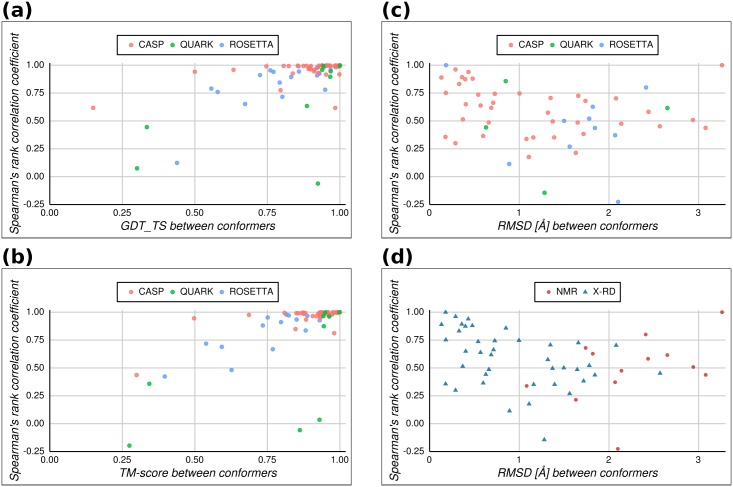
Distribution of Spearman’s rank correlation coefficients per target, computed between structural rankings for all proposed structural models against each of the conformers in the pair of maximum conformational diversity, as a function of different average measures of structural similarity between native conformers. (a) Correlation against GDT_TS between native conformers. (b) Idem (a) but using TM-score. (c) Idem (a) but using RMSD, with distinction among the three dataset used in the study (CASP 3–10, pink circles; QUARK, green circles; Rosetta@home 2007, blue circles). (b) Same as (c) but with distinction between experimental methods used for determining protein structure according to PDB (X-RD, x-ray diffraction, red circles; NMR, blue triangles).

A single structure-based approach would be promising if the target protein can be characterized by a narrow and homogeneous native state. In such cases all 3D modeling efforts should tend towards decoys that resemble the target structure closely. Nevertheless, when considering the existence of a marked conformational diversity, then the selection of protein models by their structural proximity to a unique native conformer may lead to a biased subset of structures in which decoys resembling other true conformers are not included. Hence, we explored how structurally derived rankings would be modified when different native conformers are simultaneously considered as targets in the evaluation of several 3D models proposed by various simulation protocols. For the 91 protein targets in our dataset we performed pairwise structural comparisons between the proposed decoys and each conformer in the pair with maximum conformational diversity, as identified in the previous section using CoDNaS database. When analyzing a high-quality subset (76 proteins targets) of proteins with 3 or more statistically significant structural alignments between any decoy and target pair, based on RMSD Z-scores calculated with MAMMOTH, the highest correlations occur when comparing decoys against structurally close conformers (Fig 2). As the structural distance between target conformers increase, there are more chances of finding decoys that more strongly resemble either one of them, but only if these differences could be detected. Both GDT_TS (Fig 2a) and TM-score (Fig 2b) indicate strong correlations between decoy rankings against pairs of conformers when these target conformers are very similar (e.g. when the TM-score between them exceeds 0.5[48]). While the average Spearman rank correlation coefficient is 0.89 and 0.88 using GDT_TS and TM-score respectively, it decreases to 0.55 when the RMSD is used to evaluate structural similarity (Fig 2c). Moreover, when considering how decoy rankings change depending on the structural similarity of the decoys themselves, only the RMSD can capture enough variability to explain low correlations (S4 Fig). Consequently, only when the correct structural similarity measure is used, it could be clearly noticed that the decoys are not equally distant to both known native conformations. This observation confirms that the choice of a reference structure (a given conformer) will affect the evaluation of protein 3D models.

Fig 2a–2c also discriminates the correlations by dataset, thus reflecting the different approaches used for building the structural models. It is tempting to think that template-free methods could result in a wider sampling of conformations for a given target. However, there is no clear relationship between the rank correlation coefficients and the modeling approach (the majority of CASP participants use template-based methods, while QUARK and Rosetta datasets provide template-free or ab initio models). Besides, as shown in Fig 2d, pairs of conformers determined by NMR spectroscopy are more structurally dissimilar than those obtained using x-ray crystallography, a fact that may reflect the increased exploration of solution state dynamics allowed by NMR and may explain why ranking correlations are not higher.

**Fig 3:**
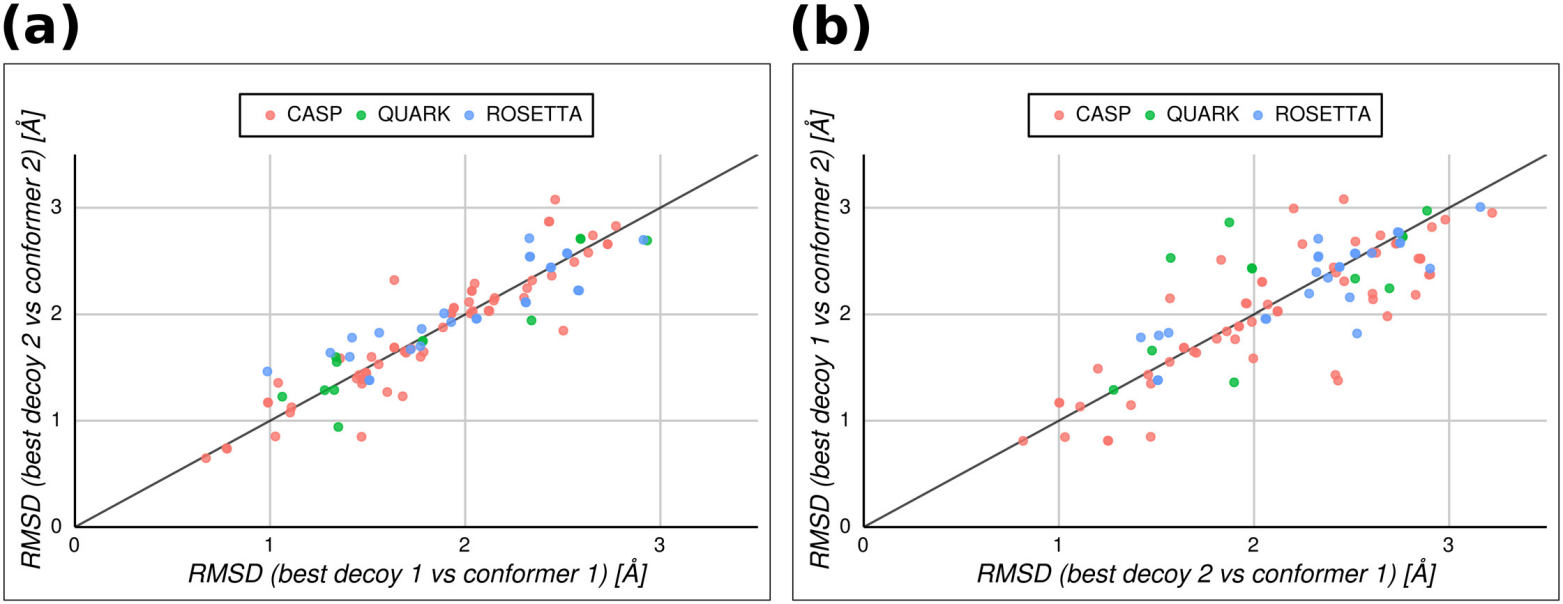
Comparison of target structure-best decoy RMSD values between both conformers of a protein showing maximum conformational diversity. (a) Distribution of decoy-target RMSD values calculated for each protein in our dataset choosing pairs of best decoys for each conformer. (b) The same comparison showed in a, but swapping conformers and best decoys. Symbols indicate the three datasets used in the study (CASP 3–10, pink circles; QUARK, green circles; Rosetta@home 2007, blue circles).

Results shown in Fig 3 give a strong support to the idea that the population of decoys contains a wide sample of structural models close to discernible conformers from the native state of a target protein. For 30% of the targets studied, the same decoy was identified as the top-ranked model using an RMSD-based comparison against conformers showing maximum RMSD (RMSD = 1.91Å on average). In these cases, the best decoy is almost equally distant to both conformers describing the putative native ensemble of the target. However, in the remaining 70% of the targets different decoys are recognized as the top-ranked ones depending on the reference conformer of choice. Best decoys in this case have a structural difference of RMSD = 1.82Å in average between the best decoys and its corresponding conformer (Fig 3a). When the best decoy against a given conformer is cross-compared with the alternative conformer of the same target, the mean RMSD increases to 2.13Å in average (Fig 3b). Here again, RMSD suggests more cases of conformational diversity than the other measures. Moreover, GDT_TS chooses the same top decoy in 49% of the datasets, or in 53% if only accepting GDT_TS equal or higher than 0.5 (as generally employed in CASP). This difference compared with RMSD is even stronger for TM-score, which recognizes a unique best model on 54% of the datasets or in 70% when filtering unreliable decoy-target pairs (those with TM-score lower than 0.5).

We also split CASP targets in ‘hard’ and ‘easy’ following CASP definition, according to their level of sequence and/or structural similarity to known folds (see e.g. [49,50]). We found that those hard targets showing two different best decoys have a mean RMSD of the best decoys against the native conformers equal to 2.26Å, while the mean RMSD for the subset of hard targets with one top decoy is even higher at 2.8Å (both are respectively higher for easy targets). Thus, the increased modeling difficulty of hard targets produces a population of decoys with worse quality. We observed that the structural similarity between conformers in both subsets of hard targets is not very different, with a mean RMSD of 1.35Å and 1.57Å for the targets with one or more best decoys, respectively. This suggests that the degree of conformational diversity may not condition an extended sampling of the native space from the proposed structural models. Instead, this may rely on how the models are generated, and specifically for homology modeling, the target-template sequence identity. In our analysis the decoys obtained with template-based methods showing a single best model were modeled on a template with relatively higher sequence identity (26.1% on average) than the putative template for targets with two best models (21.4% on average). Therefore, it is expected that by employing a wider and more divergent population of templates, homology modeling techniques as a whole could capture more of the native structural space.

### Biological insights from three-dimensional models reflecting conformational diversity

**Fig 4:**
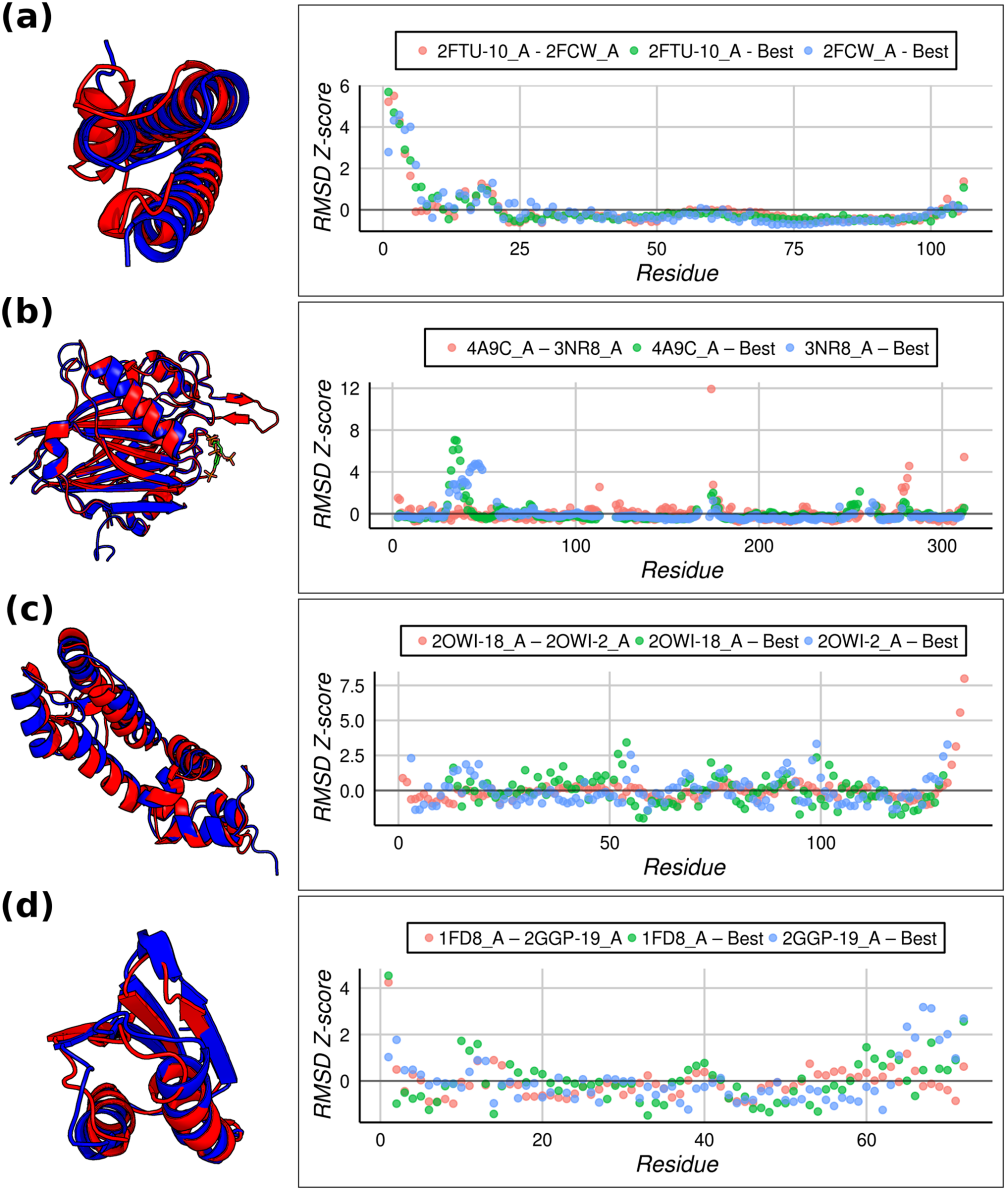
Examples of structural differences between pairs of known conformers (left panels, red and blue) and z-score distribution based on RMSD per position derived from the structural superpositions between conformers and with their corresponding best decoys (right panels). (a) Human unbound receptor associated protein (RAP) (PDB ID: 2FTU, red) and RAP bound to low-density lipoprotein receptor (LDLR) (PDB ID: 2FCW, blue). (b) Human SHIP2 protein conformers (PDB ID: 4A9C, red; PDB ID: 3NR8, blue; synthetic ligand, green). (c) NMR structures of RGS (Regulator of G protein signaling) domain of human protein RGS18 (PDB ID: 2OWI, model 2, red; PDB ID: 2OWI, model 18, blue). (d) Atx1 yeast metallochaperone in Cu(I)-bound form (PDB ID:1FD8, red) and the Atx1-Ccc2 ATPase complex (PDB ID: 2GGP, model 19, blue). See text for further details.

As previously exposed using RMSD-based comparison, for 70% of the proteins in our dataset we detected different decoys structurally closer to each of the conformers representing the target native ensemble. Fig 4 shows some examples of the structural differences between pairs of known conformers and their respective best decoys. Left panels in the figure show the structural alignment of the target conformers representing the native ensemble, while right panels present the distribution of z-scores based on RMSD per position between conformers and their corresponding best decoys. In the same figure, pink salmon dots represent the z-score coming from the conformers’ alignment while blue and green are used to represent the z-scores for the alignment between the best decoys for each conformer. We observed that regions showing structural differences are characterized by high z-scores while those more structurally similar approach zero. In Fig 4a we represent the structural alignment of conformers of the receptor-associated protein (RAP) which binds members of the low-density lipoprotein receptor (LDLR) family and facilitates their transport from the endoplasmic reticulum to the Golgi. The study of a co-crystalized structure suggests that two LDL receptor modules may be needed to stabilize RAP binding [51]. Each of these interactions is established through one (of three) conserved lysine of RAP that binds an aspartate via a salt bridge and stacks against an aromatic residue in a specialized pocket of the LDLR domain. Conformational equilibrium is displaced following protonation of conserved histidines that brings a change, at low pH, in the electrostatic surface of the RAP-D3 domain. An RMSD = 1.73Å between the unbound (PDB ID: 2FTU) and bound to LDLR (PDB ID: 2FCW) known structures of RAP-D3 reveals subtle global conformational rearrangements (Fig 4a, left). These differences can also be seen when considering the best decoys for each of the putative conformers. The decoys display an RMSD of 1.37Å and 1.57Å when compared to their closest conformer but show an increment to 2.52Å and 2.04Å respectively when cross-assessed against the other conformer. These structural similarities and differences could be appreciated by the z-scores in the right panel of Fig 4a, which evidence that alignments between the best decoy and the corresponding conformer show lower z-scores in some regions compared with equivalent z-scores derived from the alignment of conformers.

Left panel in Fig 4b shows the structural superposition between conformers of SHIP2 (SH2(Src homology 2)-domain-containing inositol-phosphatase-2). This protein is involved in intracellular signaling[52]. The double-stranded antiparallel beta sheet structure emerging on top of the conformer that was co-crystallized with a synthetic ligand (PDB ID: 4A9C) is solvent-exposed and does not appear in the free conformer (PDB ID: 3NR8) due to its high flexibility. This region plays a key role folding over the ligand to form the binding site of the protein along with other target structure poorly defined loops. However, best decoys help to understand the biological importance of these flexible regions absent in the target structure. The selected best decoys have RMSD of 1.17Å and 1.22Å against their respective targets. These values slightly increase to 1.24Å and 1.25Å respectively when they are compared against the other conformer. Additionally, Fig 4c shows two selected NMR-solved conformers of the RGS (Regulator of G protein signaling) domain of human protein RGS18 (PDB ID: 2OWI, models 2 and 18)[53]. The RGS domain family is highly heterogeneous in structures and these differences may modulate G-alpha-binding specificities. It has been shown that equilibrium displacement between conformers would selectively modulate the mechanism for RGS-G-alpha subunit complex formation, at least in the case of the homologous RGS4[54]. This plasticity is reflected in the best decoys which have an RMSD of 1.36Å and 1.59Å against their respective conformers, but are more distant from the complementary conformers at 1.57Å and 2.15Å respectively. Finally, Fig 4d illustrates the diversity of the Atx1 yeast metallochaperone protein that carries metal ions to specific sites within the cell. Analysis of the Cu(I)-bound form of Atx1 (PDB ID: 1FD8) has shown that the copper-binding cysteines become exposed on the surface of the protein after metal ion releasing [55]. This structural change also disrupts the Atx1-Ccc2 ATPase complex (PDB ID: 2GGP, model 19), a transient interaction for which copper ion is essential[56]. These conformers of Atx1 show an RMSD = 1.82Å accounting for their secondary structure arrangements (Fig 4d, left). It is also reflected by the selected best decoys, which increase the RMSD = 1.93Å to each of the conformers to 2.19Å and 2.28Å when compared with the other conformer.

## Conclusions

The increasing availability of experimental structures from native ensembles allowed us to assess the quality of protein structure models under a different perspective. We have found that considering the native state as an ensemble of conformers improves the selection of 3D structure models, thus helping to understand protein function more comprehensively. The evaluation of protein structure models is shown to be highly influenced by the conformational diversity of the target protein. This evaluation proves to be robust to the use of different structural similarity scores, in the sense that conformational diversity can always be detected if present, although with different sensibilities. Conversely, the selection of decoys is not robust regarding the choice of similarity measures, as decoy rankings would be very different.

A majority of the proteins in our dataset showing different decoys were structurally closer to alternative native ensemble conformers. This finding reveals that, although not usually taken into account, many of the current methods for predicting three-dimensional structures of proteins could be used to predict conformational diversity as well. According to our results, there is no particular tertiary structure prediction method that could stand out in the estimation of close conformers to the target. However, we did find that, when template-based methods are used, using distant evolutionary templates produces a better sampling of the conformational space than using closer homologs.

The biological analysis of several selected examples of top-ranked decoys allowed us to get a better understanding of their role as members of the native ensemble. Therefore, conformational diversity estimated from 3D modeling techniques adds an additional value for a meaningful interpretation of structure-function relationships. In the same sense, we deeply encourage the consideration of conformational diversity when conducting protein structure prediction and endorse the evaluation of conformational diversity in community-wide efforts like CASP. To this end, and while current prediction methods can be used as such, a proper selection of structural decoys that may account for conformational diversity in a target protein would necessarily require new improvements and novel methodologies of model evaluation in assessment protocols.

## Supporting Information

S1 Appendix Examples of proteins from our dataset that were used as targets in public structural modeling experiments and display conformational diversity in the native state. (DOCX)

S1 Dataset Datasets used in this study. (GZ)

S1 Fig Comparison of GDT_TS against TM-score, calculated for pairs of conformers with maximum conformational diversity for each protein in our dataset. Pairs were taken from the CoDNaS database of different structures (from available PDB files) for each represented protein. GDT_TS values are normalized to the range [0, 1].
(TIFF)

S2 Fig Comparison of RMSD against the difference in relative accessible surface area (ΔrASA), calculated for pairs of conformers with maximum conformational diversity for each protein in our dataset. Pairs of conformers were taken from the CoDNaS database for different structures. RMSD scores are expressed in Å while ΔrASA values are in Å^3^.
(TIF)

S3 Fig Distribution of cavity volume differences between pairs of conformers with maximum conformational diversity for each protein in our dataset. Pairs were taken from the CoDNaS database of different structures (from available PDB files) for each represented protein.
(TIF)

S4 Fig Distribution of Spearman’s rank correlation coefficients per target, computed between structural rankings for all proposed structural models against each of the conformers in the pair of maximum conformational diversity, as a function of different average measures of structural similarity between pairs of decoys and native conformers. (a) Correlation against average RMSD of all decoy-target pairs of each protein. (b) Same as (a) but using average GDT_TS. (C) Same as (a) but using average TM-score. (d) Correlation against the average variance of all RMSD values between decoy-target pairs of each protein. (e) Same as (d) but using average variance of GDT_TS. (f) Same as (d) but using average variance of TM-score. RMSD scores are expressed in Å while GDT_TS values are normalized to the range [0, 1].
(TIFF)

## References

[1] Tsai CJ, Kumar S, Ma B, Nussinov R (1999) Folding funnels, binding funnels, and protein function. Protein Sci 8: 1181–1190. 10.1110/ps.8.6.1181 10386868 PMC2144348

[2] Kumar S, Ma B, Tsai CJ, Sinha N, Nussinov R (2000) Folding and binding cascades: dynamic landscapes and population shifts. Protein Sci 9: 10–19. 10.1110/ps.9.1.10 10739242 PMC2144430

[3] Gunasekaran K, Ma B, Nussinov R (2004) Is allostery an intrinsic property of all dynamic proteins? Proteins 57: 433–443. 10.1002/prot.20232 15382234

[4] Changeux J-P (2012) Allostery and the Monod-Wyman-Changeux model after 50 years. Annu Rev Biophys 41: 103–133. 10.1146/annurev-biophys-050511-102222 22224598

[5] Laskowski R a, Gerick F, Thornton JM (2009) The structural basis of allosteric regulation in proteins. FEBS Lett 583: 1692–1698. 10.1016/j.febslet.2009.03.019 19303011

[6] Tobi D, Bahar I (2005) Structural changes involved in protein binding correlate with intrinsic motions of proteins in the unbound state. Proc Natl Acad Sci U S A 102: 18908–18913. 16354836 10.1073/pnas.0507603102 PMC1323175

[7] Brylinski M, Skolnick J (2008) What is the relationship between the global structures of apo and holo proteins? Proteins Struct Funct Bioinforma 70: 363–377. 10.1002/prot.21510 17680687

[8] Burra P V, Zhang Y, Godzik A, Stec B (2009) Global distribution of conformational states derived from redundant models in the PDB points to non-uniqueness of the protein structure. Proc Natl Acad Sci U S A 106: 10505–10510. 10.1073/pnas.0812152106 19553204 PMC2705611

[9] Gora A, Brezovsky J, Damborsky J (2013) Gates of Enzymes. Chem Rev 113: 5871–5923. 10.1021/cr300384w 23617803 PMC3744840

[10] Monod J, Wyman J, Changeux J-P (1965) On the nature of allosteric transitions: A plausible model. J Mol Biol 12: 88–118. 10.1016/S0022-2836(65)80285-6 14343300

[11] Henzler-wildman KA, Thai V, Lei M, Ott M, Wolf-watz M, Fenn T, et al (2007) Intrinsic motions along an enzymatic reaction trajectory. Nature 450: 838–844. doi:nature06410 [pii] 10.1038/nature06410 18026086

[12] Khersonsky O, Roodveldt C, Tawfik DS (2006) Enzyme promiscuity: evolutionary and mechanistic aspects. Curr Opin Chem Biol 10: 498–508. 10.1016/j.cbpa.2006.08.011 16939713

[13] Yogurtcu ON, Erdemli SB, Nussinov R, Turkay M, Keskin O (2008) Restricted mobility of conserved residues in protein-protein interfaces in molecular simulations. Biophys J 94: 3475–3485. 10.1529/biophysj.107.114835 18227135 PMC2292389

[14] Nussinov R, Ma B (2012) Protein dynamics and conformational selection in bidirectional signal transduction. BMC Biol 10: 2 10.1186/1741-7007-10-2 22277130 PMC3266202

[15] Juritz E, Fornasari MS, Martelli PL, Fariselli P, Casadio R, Parisi G. (2012) On the effect of protein conformation diversity in discriminating among neutral and disease related single amino acid substitutions. BMC Genomics 13 Suppl 4: S5 10.1186/1471-2164-13-S4-S5 22759653 PMC3303731

[16] Sethi A, Tian J, Derdeyn CA, Korber B, Gnanakaran S (2013) A Mechanistic Understanding of Allosteric Immune Escape Pathways in the HIV-1 Envelope Glycoprotein. 9 10.1371/journal.pcbi.1003046 23696718 PMC3656115

[17] Kopito RR, Ron D (2000) Conformational disease. Nat Cell Biol 2: 207–209. 10.1038/35041139 11056553

[18] Javier Zea D, Miguel Monzon A, Fornasari MS, Marino-Buslje C, Parisi G (2013) Protein conformational diversity correlates with evolutionary rate. Mol Biol Evol 30: 1500–1503. 10.1093/molbev/mst065 23564939

[19] Juritz E, Palopoli N, Fornasari MS, Fernandez-Alberti S, Parisi G (2012) Protein Conformational Diversity Modulates Sequence Divergence. Mol Biol Evol. doi:mss080 [pii] 10.1093/molbev/mss080 22396525

[20] Tokuriki N, Tawfik DS (2009) Protein dynamism and evolvability. Science 324: 203–207. 10.1126/science.1169375 19359577

[21] Parisi G, Zea DJ, Monzon AM, Marino-Buslje C (2015) Conformational diversity and the emergence of sequence signatures during evolution. Curr Opin Struct Biol 32: 58–65. 10.1016/j.sbi.2015.02.005 25749052

[22] Kuzu G, Gursoy A, Nussinov R, Keskin O (2013) Exploiting conformational ensembles in modeling protein-protein interactions on the proteome scale. J Proteome Res 12: 2641–2653. 10.1021/pr400006k 23590674 PMC3685852

[23] Valente AP, Miyamoto CA, Almeida FCL (2006) Implications of protein conformational diversity for binding and development of new biological active compounds. Curr Med Chem 13: 3697–3703. 17168731 10.2174/092986706779026147

[24] Palopoli N, Fornasari M, Gomez Casati D, Parisi G (2011) BEep:using BEst Evolutionary Pattern to assess 3D quality of protein structural models. Enviado Junio 2011.

[25] Kihara D, Chen H, Yang YD (2009) Quality assessment of protein structure models. Curr Protein Pept Sci 10: 216–228. 19519452 10.2174/138920309788452173

[26] Cozzetto D, Kryshtafovych A, Fidelis K, Moult J, Rost B, Tramontano A. (2009) Evaluation of template-based models in CASP8 with standard measures. Proteins 77 Suppl 9: 18–28. 10.1002/prot.22561 19731382 PMC4589151

[27] Tosatto SCE (2005) The victor/FRST function for model quality estimation. J Comput Biol 12: 1316–1327. 10.1089/cmb.2005.12.1316 16379537

[28] Konopka BM, Nebel J-C, Kotulska M (2012) Quality assessment of protein model-structures based on structural and functional similarities. BMC Bioinformatics 13: 242 10.1186/1471-2105-13-242 22998498 PMC3526563

[29] Zhang Y, Skolnick J (2004) Scoring function for automated assessment of protein structure template quality. Proteins 57: 702–710. 10.1002/prot.20264 15476259

[30] Berman HM, Westbrook J, Feng Z, Gilliland G, Bhat TN, Weissig H, et al (2000) The Protein Data Bank. Nucleic Acids Res 28: 235–242. 10.1093/nar/28.1.235 10592235 PMC102472

[31] Monzon AM, Juritz E, Fornasari S, Parisi G (2013) CoDNaS: a database of conformational diversity in the native state of proteins. Bioinformatics: 1–3. 10.1093/bioinformatics/btt405 23846747

[32] Monzon AM, Rohr CO, Fornasari MS, Parisi G (2016) CoDNaS 2.0: a comprehensive database of protein conformational diversity in the native state. Database (Oxford) 2016: baw038 –. 10.1093/database/baw038 PMC4809262 27022160

[33] Das R, Qian B, Raman S, Vernon R, Thompson J, Bradley P, et al (2007) Structure prediction for CASP7 targets using extensive all-atom refinement with Rosetta@home. Proteins 69 Suppl 8: 118–128. 10.1002/prot.21636 17894356

[34] Xu D, Zhang Y (2012) Ab initio protein structure assembly using continuous structure fragments and optimized knowledge-based force field. Proteins 80: 1715–1735. 10.1002/prot.24065 22411565 PMC3370074

[35] Ortiz AR, Strauss CEM, Olmea O (2002) MAMMOTH (matching molecular models obtained from theory): an automated method for model comparison. Protein Sci 11: 2606–2621. 10.1110/ps.0215902 12381844 PMC2373724

[36] Kabsch W, Sander C (1983) Dictionary of protein secondary structure: pattern recognition of hydrogen-bonded and geometrical features. Biopolymers 22: 2577–2637. 10.1002/bip.360221211 6667333

[37] Chothia C (1976) The nature of the accessible and buried surfaces in proteins. J Mol Biol 105: 1–12. 10.1016/0022-2836(76)90191-1 994183

[38] Sehnal D, Svobodová Vařeková R, Berka K, Pravda L, Navrátilová V, Banáš P, et al (2013) MOLE 2.0: advanced approach for analysis of biomacromolecular channels. J Cheminform 5: 39 10.1186/1758-2946-5-39 23953065 PMC3765717

[39] Le Guilloux V, Schmidtke P, Tuffery P (2009) Fpocket: an open source platform for ligand pocket detection. BMC Bioinformatics 10: 168 10.1186/1471-2105-10-168 19486540 PMC2700099

[40] Zemla A (2003) LGA: a method for finding 3D similarities in protein structures. Nucleic Acids Res 31: 3370–3374. 12824330 10.1093/nar/gkg571 PMC168977

[41] Burra P V, Zhang Y, Godzik A, Stec B (2009) Global distribution of conformational states derived from redundant models in the PDB points to non-uniqueness of the protein structure. Proc Natl Acad Sci U S A 106: 10505–10510. doi:0812152106 [pii] 10.1073/pnas.0812152106 19553204 PMC2705611

[42] Gutteridge A, Thornton J (2005) Conformational changes observed in enzyme crystal structures upon substrate binding. J Mol Biol 346: 21–28. 10.1016/j.jmb.2004.11.013 15663924

[43] Sonavane S, Chakrabarti P (2008) Cavities and atomic packing in protein structures and interfaces. PLoS Comput Biol 4: e1000188 10.1371/journal.pcbi.1000188 19005575 PMC2582456

[44] Moult J, Pedersen JT, Judson R, Fidelis K (1995) A large-scale experiment to assess protein structure prediction methods. Proteins 23: ii–v. 10.1002/prot.340230303 8710822

[45] Moult J, Fidelis K, Kryshtafovych A, Schwede T, Tramontano A (2014) Critical assessment of methods of protein structure prediction (CASP)—round x. Proteins 82 Suppl 2: 1–6. 10.1002/prot.24452 24344053 PMC4394854

[46] Kryshtafovych A, Monastyrskyy B, Fidelis K (2014) CASP prediction center infrastructure and evaluation measures in CASP10 and CASP ROLL. Proteins 82 Suppl 2: 7–13. 10.1002/prot.24399 24038551 PMC4396618

[47] Janin J (2013) The targets of CAPRI rounds 20–27. Proteins 81: 2075–2081. 10.1002/prot.24375 23900782

[48] Xu J, Zhang Y (2010) How significant is a protein structure similarity with TM-score = 0.5? Bioinformatics 26: 889–895. 10.1093/bioinformatics/btq066 20164152 PMC2913670

[49] Kinch LN, Qi Y, Hubbard TJP, Grishin N V (2003) CASP5 target classification. Proteins 53 Suppl 6: 340–351. 10.1002/prot.10555 14579323 PMC2656935

[50] Tress M, Tai C-H, Wang G, Ezkurdia I, López G, Valencia A, et al (2005) Domain definition and target classification for CASP6. Proteins 61 Suppl 7: 8–18. 10.1002/prot.20717 16187342

[51] Fisher C, Beglova N, Blacklow SC (2006) Structure of an LDLR-RAP complex reveals a general mode for ligand recognition by lipoprotein receptors. Mol Cell 22: 277–283. 10.1016/j.molcel.2006.02.021 16630895

[52] Mills SJ, Persson C, Cozier G, Thomas MP, Trésaugues L, Erneux C, et al (2012) A synthetic polyphosphoinositide headgroup surrogate in complex with SHIP2 provides a rationale for drug discovery. ACS Chem Biol 7: 822–828. 10.1021/cb200494d 22330088 PMC3355655

[53] Soundararajan M, Willard FS, Kimple AJ, Turnbull AP, Ball LJ, Schoch GA, et al (2008) Structural diversity in the RGS domain and its interaction with heterotrimeric G protein alpha-subunits. Proc Natl Acad Sci U S A 105: 6457–6462. 10.1073/pnas.0801508105 18434541 PMC2359823

[54] Moy FJ, Chanda PK, Cockett MI, Edris W, Jones PG, Mason K, et al (2000) NMR structure of free RGS4 reveals an induced conformational change upon binding Galpha. Biochemistry 39: 7063–7073. 10852703 10.1021/bi992760w

[55] Arnesano F, Banci L, Bertini I, Huffman DL, O’Halloran T V (2001) Solution Structure of the Cu(I) and Apo Forms of the Yeast Metallochaperone, Atx1. Biochemistry 40: 1528–1539. 10.1021/bi0014711 11327811

[56] Banci L, Bertini I, Cantini F, Felli IC, Gonnelli L, Hadjiliadis N, et al (2006) The Atx1-Ccc2 complex is a metal-mediated protein-protein interaction. Nat Chem Biol 2: 367–368. 10.1038/nchembio797 16732294

