# Supplementary material for: Addressing the Role of Conformational Diversity in Protein Structure Prediction
Source: PLoS One. 2016 May 9;11(5):e0154923. doi: 10.1371/journal.pone.0154923 (PMC4861349; doi:10.1371/journal.pone.0154923)
Supplement: S1 Appendix — (DOCX) [file pone.0154923.s001.docx]

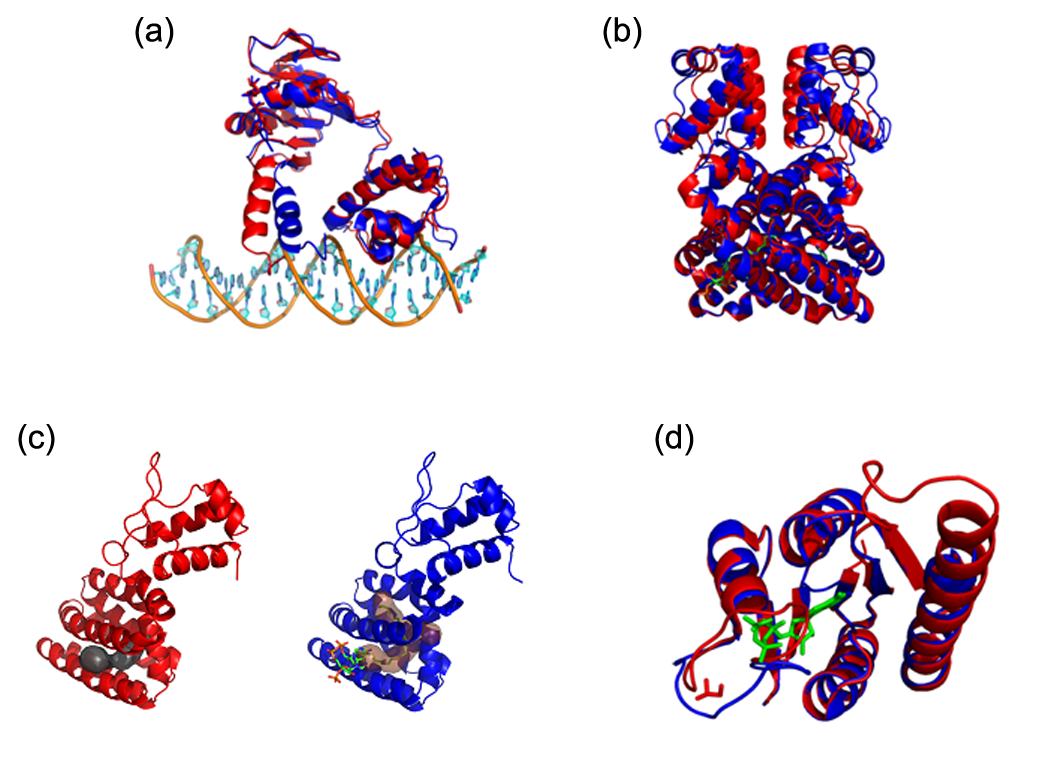


**S1 Figure. Examples of proteins from our dataset that were used as targets in public structural modeling experiments and display conformational diversity in the native state.**

(a) Superposition of the homologues T-Rex proteins from *Thermus thermophilus* (PDB ID: 3IKT_A, red) and *Thermus aquaticus* (PDB ID: 1XCB). 3IKT has been solved with NAD+ and the rex operator DNA (shown) while 1XCB was determined bound to NADH. They have identical sequences and very similar structures (RMSD=1.14Å). T-Rex is a member of the conserved Rex family of transcriptional repressors that regulate respiratory pathways following the intracellular NADH/NAD^+^ redox balance. It is a homodimer of nearly structurally identical subunits, each with an N-terminal winged helix (WH) DNA-binding domain linked via a C-terminal, “domain-swapped” α-helix to a Rossmann fold domain that binds NAD(H) cofactors. The linking α-helix also mediates formation of the T-Rex dimer[1]. The conformational change related with the molecular mechanism for sensing NADH/NAD^+^ involves a disruption of the Rex-DNA complex that favors population of a compact, “closed” dimer conformation when NADH is bound.

(b-c) Superposition of FadR conformers, a dimeric protein and transcription factor that binds acyl-Coenzyme A and regulates the expression of enzymes involved in fatty acid pathways in *Escherichia coli*. Its apo form was solved as a free dimer (PDB ID: 1E2X, red) with a DNA binding site in the N-terminal domain and a distant acyl-Coenzyme A binding site at the C-terminal domain. This conformer is able to bind DNA as a homodimer in an interaction that can be disrupted by acyl-CoA[2]. In another experimental structure (PDB ID: 1H9G, blue) FadR is bound to the effector myristoyl-CoA (myr-CoA). The structural superposition of these conformers has RMSD=1.28Å. The apo conformer shows only one tunnel and one cavity of 558Å^3^of total volume. Alternatively, the holo form, bound to myr-CoA, exhibits two more tunnels and a bigger cavity of 2144Å^3^ that facilitates ligand access to the protein core (Fig 1c). These differences highlight the importance of structural rearrangements in protein function, here separating the DNA recognition helices in the dimer and hampering DNA binding [2].

(d) Superposition of conformers of the TTHA0895 protein from *Thermus thermophilus* HB8, which belongs to the Universal stress protein family associated to enhanced cell survival. It was proposed to function as a dimer of pseudo-dimers with two subunits present in tandem in the same polypeptide chain[3]. The RMSD between its apo form (PDB ID: 2Z3V, red) and ATP-bound holo form (PDB ID: 2Z08, blue) is 1.95Å. The conformational change includes a small shift of a loop carrying an ATP binding motif[3] resulting in a better spatial organization for ligand binding.

References

1. McLaughlin KJ, Strain-Damerell CM, Xie K, Brekasis D, Soares AS, Paget MSB, et al. Structural basis for NADH/NAD+ redox sensing by a Rex family repressor. Mol Cell. 2010;38: 563–75. doi:10.1016/j.molcel.2010.05.006

2. van Aalten DMF, DiRusso CC, Knudsen J. The structural basis of acyl coenzyme A-dependent regulation of the transcription factor FadR. EMBO J. 2001;20: 2041–50. doi:10.1093/emboj/20.8.2041

3. Tkaczuk KL, A Shumilin I, Chruszcz M, Evdokimova E, Savchenko A, Minor W. Structural and functional insight into the universal stress protein family. Evol Appl. 2013;6: 434–49. doi:10.1111/eva.12057
