# Supplementary figures and images for: Addressing the Role of Conformational Diversity in Protein Structure Prediction
Source: PLoS One. 2016 May 9;11(5):e0154923. doi: 10.1371/journal.pone.0154923 (PMC4861349; doi:10.1371/journal.pone.0154923)

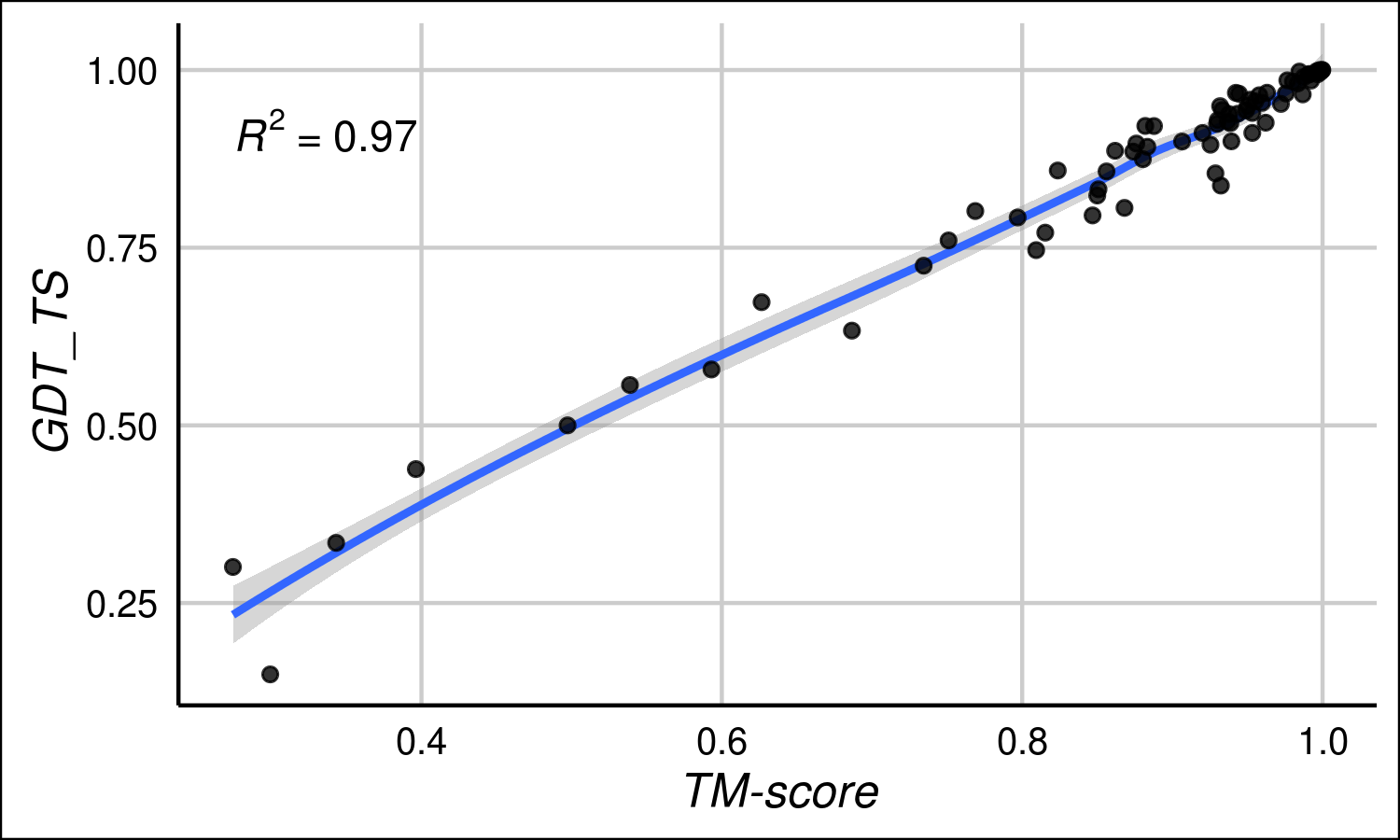

Supplement: S1 Fig — Pairs were taken from the CoDNaS database of different structures (from available PDB files) for each represented protein. GDT_TS values are normalized to the range [0, 1]. (TIFF) [file pone.0154923.s003.tiff]

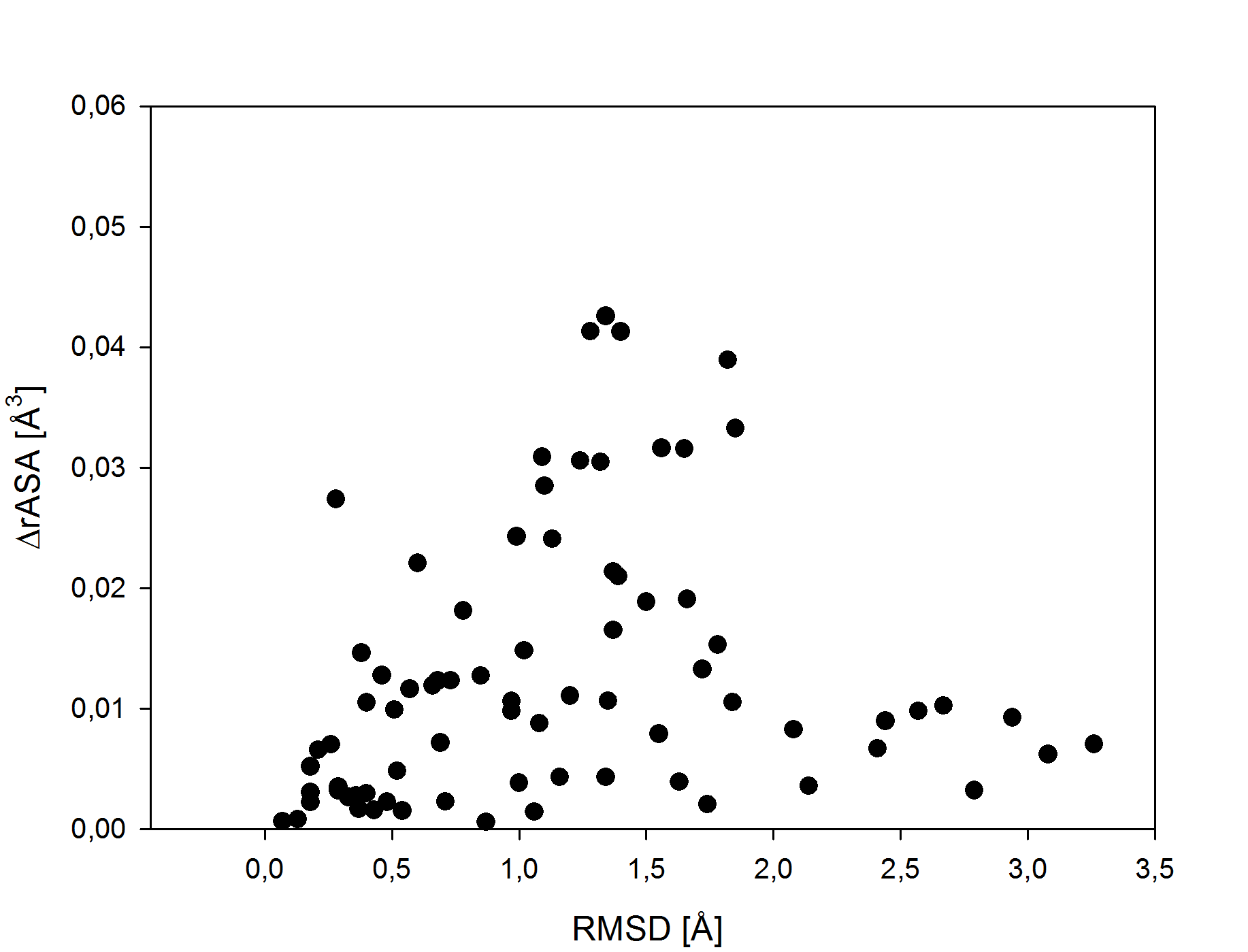

Supplement: S2 Fig — Pairs of conformers were taken from the CoDNaS database for different structures. RMSD scores are expressed in Å while ΔrASA values are in Å3. (TIF) [file pone.0154923.s004.tif]

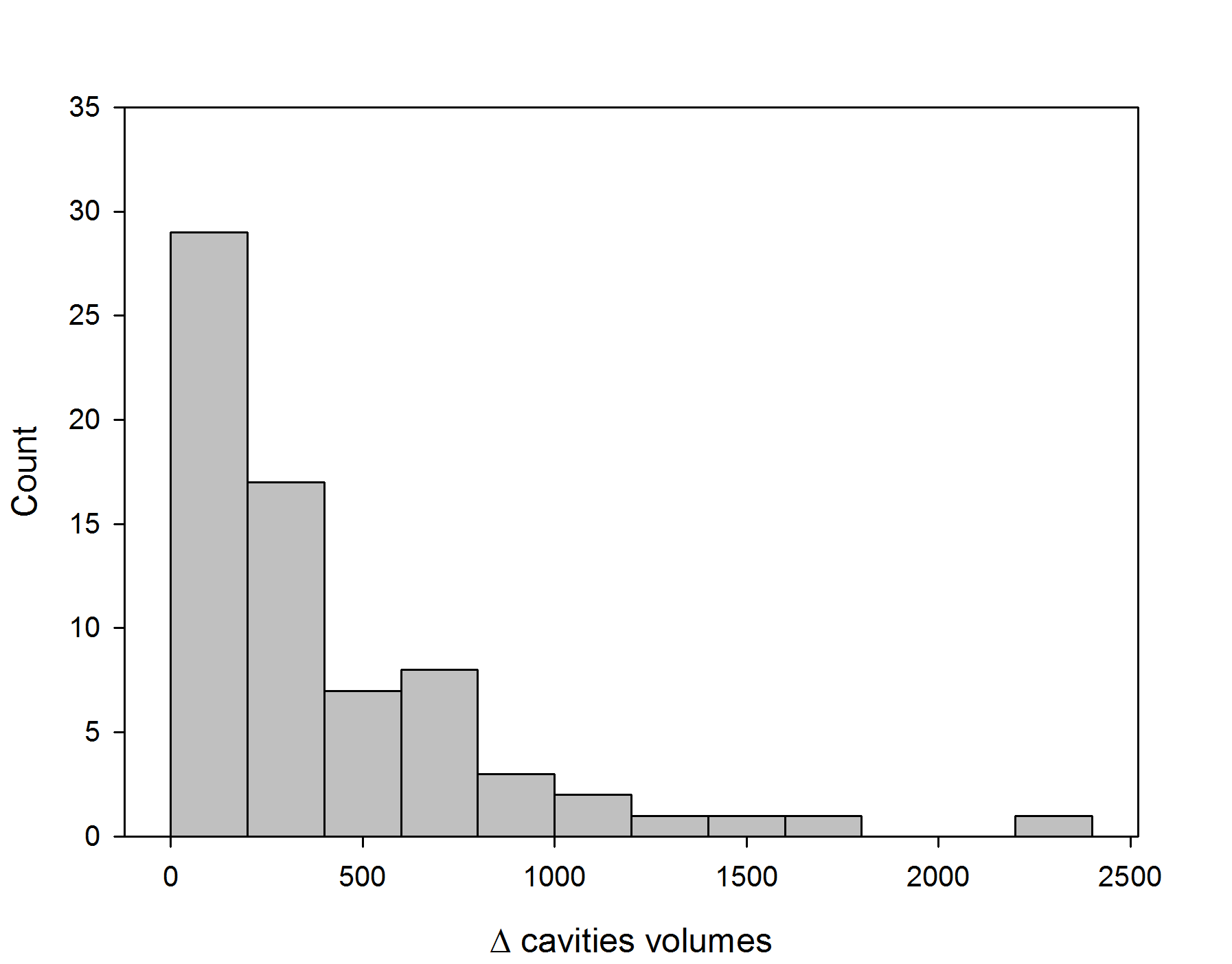

Supplement: S3 Fig — Pairs were taken from the CoDNaS database of different structures (from available PDB files) for each represented protein. (TIF) [file pone.0154923.s005.tif]

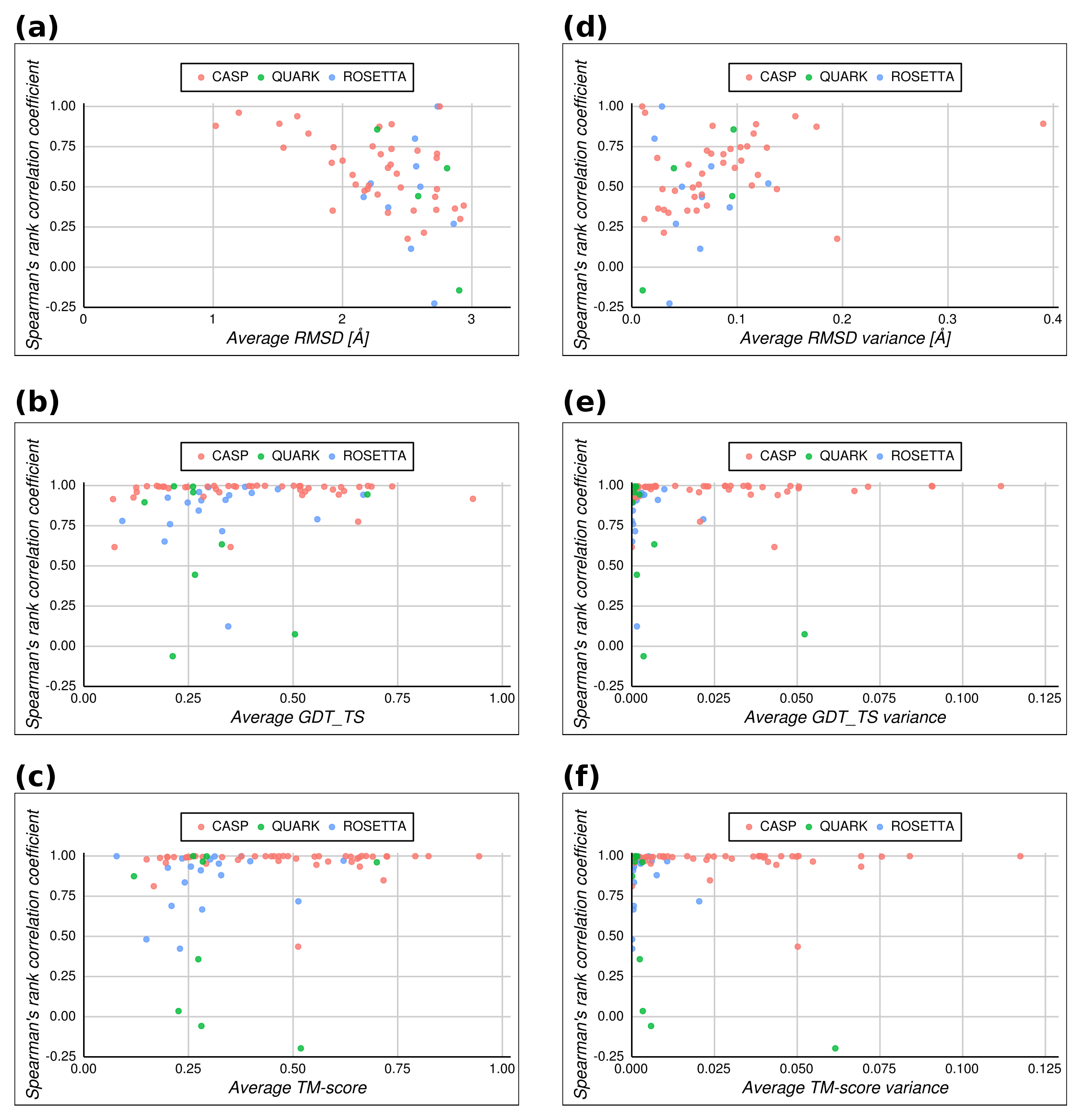

Supplement: S4 Fig — (a) Correlation against average RMSD of all decoy-target pairs of each protein. (b) Same as (a) but using average GDT_TS. (C) Same as (a) but using average TM-score. (d) Correlation against the average variance of all RMSD values between decoy-target pairs of each protein. (e) Same as (d) but using average variance of GDT_TS. (f) Same as (d) but using average variance of TM-score. RMSD scores are expressed in Å while GDT_TS values are normalized to the range [0, 1]. (TIFF) [file pone.0154923.s006.tiff]
